# The disease management program for type 2 diabetes in Germany enhances process quality of diabetes care - a follow-up survey of patient's experiences

**DOI:** 10.1186/1472-6963-10-55

**Published:** 2010-03-03

**Authors:** Ingmar Schäfer, Claudia Küver, Benjamin Gedrose, Falk Hoffmann, Barbara Ruß-Thiel, Hans-Peter Brose, Hendrik van den Bussche, Hanna Kaduszkiewicz

**Affiliations:** 1 Department of Primary Medical Care, Center of Psychosocial Medicine, University Medical Center Hamburg-Eppendorf, Martinistr 52, 20246 Hamburg, Germany; 2 Centre for Social Policy Research, Division Health Economics, Health Policy and Outcomes Research, University of Bremen, Ausser der Schleifmuehle 35-37, 28203 Bremen, Germany; 3 ife Gesundheits-AG, Gut Nehmten, 24326 Nehmten, Germany; 4 Department of Medical Biometry and Epidemiology, Center for Experimental Medicine, University Medical Center Hamburg-Eppendorf, Martinistr 52, 20246 Hamburg, Germany

## Abstract

**Background:**

In summer 2003 a disease management program (DMP) for type 2 diabetes was introduced on a nationwide basis in Germany. Patient participation and continuity of care within the DMP are important factors to achieve long-term improvements in clinical endpoints. Therefore it is of interest, if patients experience any positive or negative effects of the DMP on their treatment that would support or hamper further participation. The main objective of the study was to find out if the German Disease Management Program (DMP) for type 2 diabetes improves process and outcome quality of medical care for patients in the light of their subjective experiences over a period of one year.

**Methods:**

Cohort study with a baseline interview and a follow-up after 10.4 ± 0.64 months. Data on process and outcome measures were collected by telephone interviews with 444 patients enrolled and 494 patients not enrolled in the German DMP for type 2 diabetes. Data were analyzed by multivariate logistic regression analyses.

**Results:**

DMP enrolment was significantly associated with a higher process quality of care. At baseline enrolled patients more often reported that they had attended a diabetes education course (OR = 3.4), have ≥ 4 contacts/year with the attending physician (OR = 3.3), have at least one annual foot examination (OR = 3.1) and one referral to an ophthalmologist (OR = 3.4) and possess a diabetes passport (OR = 2.4). Except for the annual referral to an ophthalmologist these parameters were also statistically significant at follow-up. In contrast, no differences between enrolled and not enrolled patients were found concerning outcome quality indicators, e.g. self-rated health, Glycated hemoglobin (GHb) and blood pressure. However, 16-36% of the DMP participants reported improvements of body weight and/or GHb and/or blood pressure values due to enrolment - unchanged within one year of follow-up.

**Conclusions:**

In the light of patient's experiences the DMP enhances the process quality of medical care for type 2 diabetes in Germany. The lack of significant differences in outcome quality between enrolled and not enrolled patients might be due to the short program duration. Our data suggest that the DMP for type 2 diabetes should not be withdrawn unless an evidently more promising approach is found.

## Background

Health care improvement for patients with type 2 diabetes is an important target of health care policy in Germany. In summer 2003 a disease management program (DMP) for type 2 diabetes was introduced on a nationwide basis within the statutory health insurance, which covers 86% of the German population. Participation in the DMP is voluntary for physicians and patients and connected with financial incentives for both provided by the statutory health insurances. These in turn promote participation heavily as they also obtain a significant financial benefit for every enrolled patient [1]. According to latest data in January 2006 75% of the general practitioners (GPs) in Germany participated in the DMP [2]. 6 years after the start of the DMP, in August 2009, approximately 64% of the estimated five million compulsorily insured patients with type 2 diabetes were enrolled into the program [3]. Medical services in the DMP include a defined frequency of visits to the attending physician, rules for referral to a diabetologist, regular foot and eye examinations, physician counseling regarding lifestyle changes (e.g. nutrition, smoking, exercise), participation in diabetes education courses and agreement on target values for Glycated hemoglobin (GHb) and blood pressure between physician and patient. Further elements of the program are the documentation of the course of disease and treatment every 3-6 months, reminders for physicians and patients and a continuous evaluation of the DMP [4].

A description of the history and design of the German DMP is found in various papers [1,4-6]. The introduction of the DMP in Germany was strongly criticized by physicians associations and other organizations. It was forwarded that evidence for the effectiveness of the program was lacking and that the official program evaluation was not methodologically sound due to the lack of a control group [7]. Till today, the effectiveness of the DMP has not yet been satisfactorily demonstrated. In the meantime, an analysis of health insurance claims data comparing enrolled and not enrolled patients indicates that process quality of diabetes care is better for enrolled patients [8]. Concerning outcome quality there is no evidence of effectiveness, yet. It is assumed that patient participation and continuity of care within the DMP are important factors to achieve long-term improvements in clinical endpoints. Therefore it is of interest, if patients experience any positive or negative effects of the DMP on their treatment that would support or hamper further participation. Furthermore before the introduction of the DMP many doctors feared a constriction of their therapeutic freedom resulting in a loss of the possibility to treat patients individually with negative consequences on the patient-doctor relationship [9]. It is also of interest, if these fears became true. The only published study examining the subjective experiences in diabetes care of enrolled and not enrolled patients showed manifold differences between the groups pointing to a better care for enrolled patients [10], but the differences between the groups were small and - with one exception - the analyses were not controlled for important confounders, e.g. the duration of diabetes disease or the presence of depressive symptoms. The only multivariate analysis with health satisfaction as outcome emphasizes how important this would have been. The analysis shows that besides being a DMP participant also male sex, higher school education, older age (70-79 vs. 45-59 years), and a self-rated low severity of diabetes disease were significantly associated with higher health satisfaction [11]. Changes in the subjective experiences of patients over time were not investigated [10,11].

Thus, the main objective of the study was to find out if in the view of patients with type 2 diabetes the German DMP improves medical care over the period of one year. This objective is related to both process and outcome quality. We presumed that both, process and outcome quality would be better in enrolled than in not enrolled patients.

In addition we wanted to know, if enrolled patients experience benefits which they directly attribute to the DMP and we wanted to learn about the attitudes of not enrolled patients towards enrolment. Finally, we were interested to learn if certain groups of patients experience more benefits than others.

## Methods

To assess differences between diabetes care within the German DMP for type 2 diabetes and practice as usual we performed telephone interviews with enrolled and not enrolled patients twice with a follow-up period of 10.4 ± 0.64 months. Concerning process quality we investigated whether according to the experiences of the patients the medical services defined in the DMP (e.g. education courses for diabetes, referrals to an ophthalmologist etc.) were delivered by the attending physicians.

Regarding outcome quality both data on subjective outcomes (e.g. self rated health, treatment satisfaction and burden of therapy) and objective diabetes related parameters (GHb, blood pressure, body mass index, foot lesions, present symptoms of diabetes, smoking status, and hypoglycaemia in the past 12 months) were collected and compared.

All collected data on indicators of process and outcome quality are listed in Table 1. In addition patient reported data were also collected on:

◦ the duration of diabetes in months/years,

◦ depression (using a screening test based on PHQ-2 questionnaire [12], resulting in a score of 0-6, with ≥ 3 points raising suspicion of depression),

◦ the specialty of the regularly attending physician (GP or diabetologist),

◦ the type of diabetes specific medication (insulin with or without oral medication, oral medication only or no medication at all),

◦ the patient's level of education according to the CASMIN classification [13],

◦ the interviewer-rated German language skills of the patients and

◦ age and gender.

**Table 1:** Descriptive analysis of patient reported process and outcome indicators at baseline and follow-up

Process indicators	DMP-patients (baseline)	Patients not enrolled (baseline)	*p *(baseline)	DMP-patients (follow-up)	Patients not enrolled (follow-up)	*p *(follow-up)
Participation in training program for diabetes	322 (72.7%)	222 (45.1%)	p ≤ 0.001	250 (71.8%)	181 (52.8%)	p ≤ 0.001

≥4 encounters/year with physician	398 (98.8%)	361 (75.5%)	p ≤ 0.001	321 (92.5%)	280 (82.8%)	p ≤ 0.001

Annual referral to an ophthalmologist	411 (93.0%)	380 (78.2%)	p ≤ 0.001	318 (92.4%)	281 (83.9%)	p ≤ 0.001

≥1 annual referral to a diabetologist (GP patients only)	43 (13.1%)	44 (10.7%)	n.s.	24 (9.6%)	26 (9.4%)	n.s.

Specialty of the attending physician: diabetologist	113 (25.5%)	73 (14.9%)	p ≤ 0.001	100 (28.6%)	64 (18.6%)	p ≤ 0.01

Annual foot examination	380 (86.2%)	324 (66.9%)	p ≤ 0.001	307 (88.2%)	246 (73.2%)	p ≤ 0.001

Possession of a diabetes passport	345 (78.1%)	287 (59.1%)	p ≤ 0.001	276 (79.3%)	210 (61.8%)	p ≤ 0.001

Nutritional advice given by physician	116 (29.6%)	98 (23.0%)	p ≤ 0.05	71 (25.2%)	69 (26.7%)	n.s.

Physician advice given on exercising	116 (32.9%)	80 (22.5%)	p ≤ 0.01	79 (33.1%)	66 (30.1%)	n.s.

Agreement upon target value for GHb	316 (77.3%)	295 (72.8%)	p ≤ 0.05	244 (79.0%)	219 (79.3%)	n.s.

Agreement upon target values for blood pressure	326 (78.2%)	339 (76.9%)	n.s.	255 (76.6%)	232 (76.6%)	n.s.

Participation in education course for hypertension	50 (11.3%)	33 (6.7%)	p ≤ 0.05	43 (12.3%)	33 (9.7%)	n.s.

**Outcome indicators**						

Low self-rated health (1-4 on a 6-point scale)	171 (38.6%)	215 (43.7%)	n.s.	149 (42.6%)	147 (42.6%)	n.s.

(1-4 on a 6-point scale)	64 (14.5%)	74 (15.4%)	n.s.	52 (14.8%)	60 (17.5%)	n.s.

High burden of therapy (5-6 on a 6-point scale)	3 (0.7%)	11 (2.2%)	n.s.	4 (1.1%)	4 (1.2%)	n.s.

High self-rated ability to cope with disease (5-6 on a 6-point scale)	419 (94.8%)	445 (91.9%)	n.s.	319 (92.2%)	319 (93.3%)	n.s.

High self-reported therapy adherence (5-6 on a 6-point scale)	323 (72.9%)	349 (70.9%)	n.s.	244 (69.7%)	240 (70.0%)	n.s.

Mean GHb (%)	6.9 (SD: 0.9; n = 330)	6.9 (SD: 1.3; n = 287)	n.s.	6.9 (SD: 0.9; n = 245)	6.8 (SD:1.1; n = 200)	n.s.

high GHb [> 7.5% (> 8.5% for age 75+)]	72 (21.8%)	52 (18.1%)	n.s.	46 (18.8%)	35 (17.5%)	n.s.

Mean blood pressure (mm Hg)	132/79 (SD: 14.0/8.7; n = 360)	135/80 (SD: 14.9/9.1; n = 398/397)	p ≤ 0.05	133/80 (SD: 13.9/8.2 n = 293)	133.9/81 (SD: 14.3/9.5 n = 276)	n.s.

high blood pressure (≥ 140/90 mm Hg)	137 (38.1%)	186 (46.7)	p ≤ 0.05	129 (44%)	116 (42%)	n.s.

Mean body mass index (kg/m^2^)	29.7 (SD: 4.8; n = 440)	29.4 (SD: 5.3; n = 482)	n.s.	29.5 (SD: 9.1 n = 351)	28.3 (SD: 10.9 n = 342)	n.s.

high BMI (BMI ≥ 30 kg/m^2^)	188 (42.7%)	191 (39.6%)	n.s.	149 (42.5%)	137 (40.1%)	n.s.

Diabetic foot lesions	45 (10.2%)	49 (10.0%)	n.s.	29 (8.3%)	29 (8.5%)	n.s.

Present symptoms of diabetes	153 (36.1%)	173 (35.8%)	n.s.	108 (31.1%)	126 (36.8%)	n.s.

Current smoker	74 (16.7%)	82 (16.6%)	n.s.	56 (16.0%)	61 (17.7%)	n.s.

Hypoglycemia in the past 12 months	26 (5.9%)	29 (6.0%)	n.s.	23 (6.6%)	12 (3.6%)	n.s.

Insulin treatment	153 (34.5%)	129 (26.3%)	p ≤ 0.01	130 (37%)	107 (31%)	n.s.

Increased cardiovascular risk [GHb ≥ 7.5% (≥ 8.5% for age 75+) and/or blood pressure ≥ 140/90 mm Hg]	186 (56.9%)	221 (66.2%)	≤0.05	147 (56.5%)	136 (60.2%)	n.s.

Not knowing GHb test results	72 (16.3%)	166 (33.9%)	p ≤ 0.001	59 (16.9%)	97 (28.3%)	p ≤ 0.001

Not knowing blood pressure values	39 (8.8%)	50 (10.1%)	p ≤ 0.05	13 (3.7%)	30 (8.7%)	p ≤ 0.01

n.s. = statistically not significant (p > 0.05)

**Figure 1:**
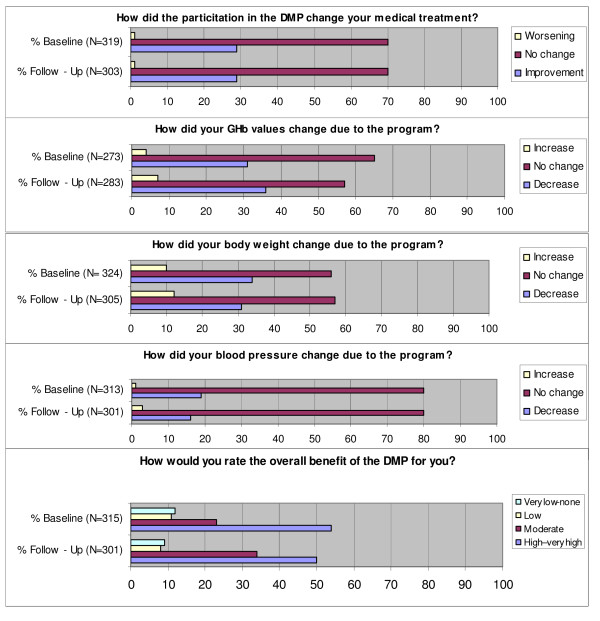
Patient-reported benefits of treatment in a DMP (enrolled patients only*). * Variations in the sample size of 444 enrolled patients at baseline and 351 enrolled at follow-up are due to 1) patients, who formally had been enrolled in the program, but did not remember this in the interview and 2) patients, whose enrollment data at timepoint of the interview were wrong and who therefore were not asked these questions.

Furthermore, enrolled patients were asked to assess the benefits of the DMP experienced by them (the questions asked are listed in Figure 1) and not enrolled patients were asked about their attitude towards enrolment in a DMP.

### Patient recruitment

Probands were selected from the database of the Gmünder ErsatzKasse (GEK), a statutory health insurance nationwide operating for 1.5 million members. Inclusion criteria for the study were a medical diagnosis of type 2 diabetes (defined by an ICD-10 code of E11, E12 or E14) by a primary care physician or a specialist and a minimum age of 40 years in order to minimize the inclusion of patients with type 1 diabetes. In addition, the "enrolled" patients had to be enrolled in the DMP for at least six months at time of the interview. The not enrolled patients in our survey should have never been enrolled in the DMP. The patients were randomly selected from the database in a consecutive way, contacted by telephone and - if possible - interviewed. Interviews were performed by a professional medical call-center (ife Gesundheits-AG, located in Nehmten). All interviewers were physicians instructed by members of the study group.

**Figure 2:**
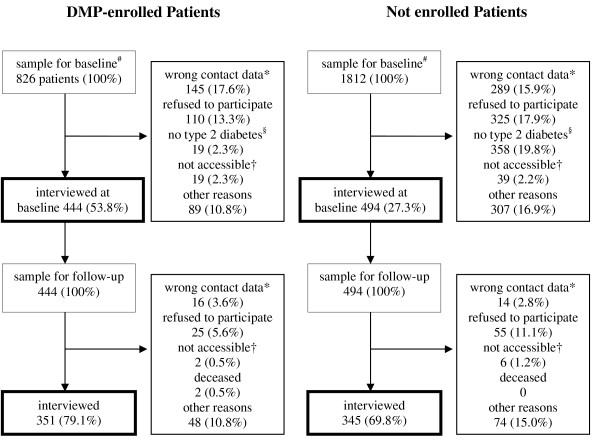
**Description of sampling and response rate for baseline and follow-up**. * wrong contact data, although the telephone numbers of the insurants were updated using accessible public databases. † not accessible = not personally accessible despite 10 attempts with defined time intervals of up to 7 days. ^# ^138 further patients were in the baseline sample but could not be assigned to any group when more detailed enrollment data were available in the course of data analysis. 57 of these patients were interviewed at baseline and 39 reinterviewed at follow-up. Their interviews were not included into the analyses. ^§ ^according to patient; among enrolled patients: 14 (1,7%) no diabetes, 5 (0,6%) diabetes type 1; among not enrolled patients: 307 (16,9%) no diabetes, 51 (2,8%) diabetes type 1.

In total 2776 patients were contacted for the baseline interview and 995 patients were interviewed (500 enrolled and 495 not enrolled in a DMP). In the course of the project more detailed data on the enrolment status of the patients became available from the insurance company, including the exact enrollment date. In order to assure inclusion quality, the initial assignment of patients to the enrolment status was revised. The revision resulted in an assignment of 444 interviewed patients to the group of enrolled patients and 494 to the group of not enrolled patients. In sum 938 patients were included in the analyses. 138 contacted patients could not be assigned to any group as they did not fulfill the inclusion criteria at baseline any more. These were patients whose participation in the DMP had finished or patients who had been participating in the DMP for less than 6 months at timepoint of interview. At follow-up approximately one year later (mean 10.4 months, SD 0.64, range 8-13 months), 696 (74.2%) of the 938 patients could be interviewed again. These 696 patients were included into the follow-up analyses due to an intention to treat approach, i.e. also participants who had changed groups at follow-up were analyzed according to their assignment at baseline. Figure 2 shows the patient-flow through the study.

### Non-responder analysis

All patients selected from the database of the GEK who were not interviewed were considered as non-responders. Based on the GEK claims data no significant differences between responders (interviewed patients; n = 995) and non-responders (n = 1781) regarding age, sex and most diabetes related diagnoses (glomerular disorders, chronic renal failure, peripheral angiopathy, disorders of lipoprotein metabolism, chronic stroke, chronic ischemic heart disease, and heart failure) were found.

In the group of responders, slightly more patients suffered from hypertension (80.4 vs. 71.6%; p ≤ 0.001), diabetic retinopathy (9.6 vs. 6.9%; p ≤ 0.01) and were insulin-dependent (29.5 vs. 20.7%; p ≤ 0.001), but stayed fewer days in hospital (mean total days in hospital: 3.0 ± 8.8 vs. 3.8 ± 11.2; p ≤ 0.05). Furthermore, responders had received more prescriptions of different drugs within one year (mean total number of different drug prescriptions: 8.3 ± 4.8 vs. 7.5 ± 5.2; p ≤ 0.001), which can be interpreted as a gross indicator of greater morbidity. In short, the non-responder analysis showed that an explicit selection bias in the direction of a greater morbidity of non-responders did not occur.

### Ethical approval

The study was approved by the Ethics Committee of the Chamber of Physicians of Hamburg (reference number OB-008/07).

### Statistical Analyses

For the description of the sample, univariate tests of significance (t-test and chi-square test) were performed. In these analyses we applied the usual significance levels of p ≤ 0.05, p ≤ 0.01 and p ≤ 0.001. In addition, process and outcome indicators of enrolled and not enrolled patients were compared by multivariate logistic regression analyses. Odds ratios (OR) for all variables that reached the p ≤ 0.001 significance level were calculated. Age, sex, education, German language skills, duration of diabetes and signs of depression were used as control variables. We also calculated differences in high risk status between enrolled and not enrolled patients. A high risk status was assumed if a patient had GHb ≥ 7.5% (≥ 8.5% for patients ≥ 75 years) and/or blood pressure ≥ 140/90 mmHg. These criteria were chosen according to the "GP's handbook" [14], the official national evidence-based guideline for the type 2 diabetes DMP.

Multivariate logistic regression analyses with the above mentioned control variables were also performed for an analysis of the association of patient characteristics with patient-reported benefits of treatment in a DMP and the attitude towards enrolment in not enrolled patients. In addition the duration of DMP participation was included as control variable in the analyses with enrolled patients, as it could influence the perceived benefits of treatment.

For each statistical model we used backward selection algorithms (based on likelihood ratio) to identify interactors (effect modifiers) within all possible two-factor terms of exposure and control variables (p ≤ 0.005 for exclusion; p ≤ 0.001 for re-inclusion). Interactors were excluded stepwise if they had no significant effect (p ≤ 0.001) after adjusting for all control variables. The final statistical analyses contained all exposure variables, all control variables and significant interactors. For the analysis of the follow-up data, the raw score of the dependent variable at baseline was included as another control variable, where appropriate.

As the data analyses are mainly explorative, we used rigorous criteria to minimize the number of false positive results. For all multivariate logistic regression analyses an α-level of 0.1% (p ≤ 0.001) was defined as statistically significant. All statistical tests were conducted with SPSS 16.0.

## Results

In our sample DMP and usual care patients did not differ in age, gender, level of education and German language skills. Also, there was no difference between enrolled and not enrolled patients concerning the duration of diabetes (see Table 2).

**Table 2:** Sociodemographic data of enrolled and not enrolled patients at baseline

	Patients enrolled in DMP (N = 444)	Patients not enrolled (N = 494)	p
Age mean (SD)	63.8 (8.49)	63 (10.1)	n.s.

Men (%)	275 (61.9%)	302 (61.1%)	n.s.

Level of Education:	N = 420	N = 474	n.s.
- high (%)	276 (65.7%)	325 (68.6%)	
- middle (%)	107 (25.5%)	111(23.4%)	
- low (%)	37 (8.8%)	38 (8.0%)	

Inferior German language skills (%)	30 (6.8%)	28 (5.7%)	n.s.

Mean duration of diabetes (in years) (SD)	N = 437 8.6 (7.45)	N = 485 8.04 (6.88)	n.s.

The DMP-effect was assessed by comparing process and outcome indicators between enrolled and not enrolled patients for baseline and follow-up. Descriptive data are shown in Table 1, the results of the multivariate logistic regression analyses are presented in Table 3.

**Table 3:** Association of enrolment in the DMP with patient reported process and outcome indicators, results of the logistic regression analyses

	Baseline			Follow-up		
**Process indicators**	**Odds Ratio (95% confidence interval)**	**Significant confounders**	**R^2^**	**Odds Ratio (95% confidence interval)**	**Significant confounders**	**R^2^**

Participation in education course for diabetes	3.4 (2.5-4.6)	diabetes duration (longer)	0.173	2.1 (1.5-3.0)	diabetes duration (longer)	0.118

≥4 encounters/year with physician	3.3 (2.2-5.1)	None	0.097	3.1 (1.8-5.3)	None	0.071

Annual referral to an ophthalmologist	3.4 (2.2-5.4)	Depr. score (lower)	0.134	n.s.	None	0.066

≥1 referral to diabetologist/year (GP patients only)	n.s.	Age (younger)	0.124	n.s.	None	0.024

Specialty of the attending physician: diabetologist	2.0 (1.4-2.9)	diabetes duration (longer)	0.090	n.s.	None	0.048

Annual foot examination	3.1 (2.2-4.4)	None	0.083	3.2 (2.0-4.9)	None	0.082

Possession of a diabetes passport	2.4 (1.7-3.2)	diabetes duration (longer)	0.110	2.1 (1.5-3.0)	None	0.058

Nutritional advice given by physician	n.s.	None	0.021	n.s.	None	0.032

Physician advice given on exercising	n.s.	Age (younger)	0.071	n.s.	None	0.030

Agreement on target value for GHb	n.s.	Age (younger)	0.092	n.s.	Age (younger)	0.091

Agreement upon target values for blood pressure	n.s.	None	0.015	n.s.	None	0.023

Participation in education course for hypertension	n.s.	None	0.025	n.s.	None	0.018

**Outcome Indicators**						

Self-rated health (S-rh) (low)	n.s.	Depr. score (higher)	0.165	n.s.	Depr. score (higher), S-rh at t0 (lower)	0.200

Satisfaction with medical treatment (low)	n.s.	None	0.028	n.s.	Satisf. with med. treatment t0 (lower)	0.119

Burden of therapy (high)	n.s.	None	0.125	n.s.	None	0.078

Self-rated ability to cope with disease (high)	n.s.	Depr. score (lower), language skills (better)	0.120	n.s.	Depr. score (lower)	0.017

Self-reported therapy adherence (S-rta) (high)	n.s.	Age (older), language skills (better)	0.080	n.s.	S-rta at t0 (higher), Depr. score (lower)	0.138

GHb [≥ 7.5% (≥ 8.5% for age 75+)]	n.s.	diabetes duration (longer)	0.070	n.s.	GHb level at t0 (high)	0.365

Blood pressure (≥ 140/90 mm Hg)	n.s.	None	0.052	n.s.	Blood pressure at t0 (high)	0.231

Body mass index (≥ 30 kg/m^2^)	n.s.	Age (younger), sex (female)	0.067	n.s.	BMI at t0 (high)	0.726

Diabetic foot lesions (Dfl)	n.s.	Depr. score (higher)	0.073	n.s.	Dfl at t0, depr. score (higher)	0.193

Present symptoms of diabetes	n.s.	Depr. score (higher)	0.144	n.s.	Depr. score (higher)	0.117

Smoking	n.s.	Age (younger)	0.157	n.s.	Status as smoker at t0	0.808

Risk of hypoglycemia (high)	n.s.	None	0.079	n.s.	None	0.163

Insulin treatment	n.s.	diabetes duration (longer)	0.227	n.s.	Insulin treatment at t0	0.839

Increased cardiovascular risk*	n.s.	Education (lower)	0.097	n.s	Increased cardiovascular risk at t0	0.262

Knowing GHb test results	2.8 (2.0-4.0)	age (younger), language skills (better), diabetes duration (longer)	0.186	n.s.	Knowing GHb test results at t0	0.289

Knowing blood pressure test results	n.s.	None	0.034	n.s.	Knowing blood pressure at t0	0.216

Multivariate logistic regression analyses. Enrolled patients compared to not enrolled patients adjusted for age, sex, education, duration of diabetes disease, German language skills and depressive symptoms. In addition outcome indicators at follow-up controlled for baseline values (t0). Direction of association is indicated in brackets; p ≤ 0.001 for all odds ratios shown as numerical values; n.s. = statistically not significant (p > 0.001). *Increased cardiovascular risk = [GHb ≥ 7.5% (≥ 8.5% for age 75+) and/or blood pressure ≥ 140/90 mm Hg]; Depr. = Depression

### Differences in process and outcome indicators at baseline

Regarding process measures we found that enrolment in the DMP was significantly associated with a more frequent utilization of type 2 diabetes-specific medical services. Enrolled patients more often

- had participated in training programs for diabetes,

- had ≥ 4 encounters/year with their physician,

- had at least one annual referral to an ophthalmologist,

- reported that the mainly attending physician was a diabetologist,

- had at least one annual foot examination and

- possessed a diabetes passport (all p ≤ 0.001).

These differences were statistically significant both in the descriptive and the multivariate logistic regression analyses. Other differences regarding process measures disappeared in the multivariate analyses after adjusting for selected control variables. This holds for the frequency of nutritional advice and advice on exercising by their physician, the frequency of agreement on a target value for GHb and the participation in educational trainings for hypertension.

Aside being enrolled in the DMP the duration of the diabetes was associated with three process measures: The longer the patients had their diagnosis of diabetes, the greater was the probability of having participated in a training program for diabetes, of being mainly treated by a diabetologist and possessing a diabetes passport. Three other indicators of process quality were not associated with DMP enrollment, but associated with age: younger patients more often received a referral to a diabetologist/year, more often got an advice on exercising and more often had agreed on a target value for GHb than older patients.

In contrast to the measures of process quality, no differences between enrolled and not enrolled patients were found for the outcome measures self-rated health, treatment satisfaction, burden of therapy, self-rated adherence to medical therapy and the ability to cope with the disease. Also there were no differences for GHb, body mass index, smoking status, hypoglycemia in the past 12 months, frequency of foot lesions and presence of diabetic symptoms, neither in the univariate nor in the multivariate analyses.

In the univariate analyses self-reported blood pressure was slightly higher in not enrolled patients (135/80 vs. 132/79 in enrolled patients), more of the enrolled patients were treated with insulin (34.5 vs. 26.3%), and less of the enrolled had an increased cardiovascular risk (56.9 vs. 66.2%), but these differences disappeared in the multivariate analyses. However, a higher percentage of not enrolled patients did not know their test results for GHb (33.9 vs. 16.3%; p ≤ 0.001) and blood pressure (10.1 vs. 8.8%; p ≤ 0.05). Not knowing GHb test results was the only outcome difference that was statistically significant both in the univariate and in the multivariate analyses at baseline.

A higher depression score was a significant confounder concerning more subjectively coloured outcome indicators. A high depression score was associated with low self-rated health, the perception of present diabetes symptoms, the perceived presence of diabetic foot lesions and a low self rated ability to cope with the disease. Also, age was a significant confounder: older age was associated with a higher self-reported adherence to therapy, not knowing the GHb test results, not smoking and a lower body-mass index.

### Differences in process and outcome indicators at follow-up

The analysis of the follow-up data confirmed the results of the baseline. In the multivariate analyses differences between enrolled and not enrolled patients were only found for parameters of process quality. Enrolled patients more often

• had participated in training programs for diabetes,

• had at least 4 encounters/year with their physician,

• had at least one annual foot examination and

• possessed a diabetes passport.

Compared to the baseline, the difference between enrolled and not enrolled patients with regard to at least one annual referral to an ophthalmologist and being mainly treated by a diabetologist did not reach statistical significance. Interestingly most of the outcome measures at follow-up were strongly associated with the respective outcome measures at baseline.

### Patient-reported benefits of treatment in a DMP

Enrolled patients were asked whether their participation in the DMP had resulted in a change of their medical treatment for diabetes. At baseline and at follow-up, the majority of DMP participants did not report any change (70%), whereas 29% reported that their treatment had improved due to enrolment. Approximately one third of the enrolled indicated a decrease of their GHb and a decrease of body weight due to enrolment - at both interviews. 19% at baseline and 16% at follow-up reported a decrease of blood pressure. Asked for a global rating of perceived benefit of the DMP, the patients' opinion was very positive: 54% of the patients at baseline and 50% at follow-up saw a high to very high benefit on a 6 point scale, 23% (and 34% at follow-up) a moderate benefit. About one fifth of enrolled patients perceived low, very low or no benefit of the program at both interviews (see Figure 1).

Analyzing the association of patient socio-demographic characteristics with reported improvements of treatment due to DMP enrolment at baseline, we found that a low depression score was associated with reporting a general improvement of the medical treatment due to enrolment (OR 1.4 for each point less in the depression score, 95% CI 1.02-2.0, p ≤ 0.05). Female gender was associated with reporting an improvement of GHb test results (OR 2.5, 95% CI 1.4-4.6, p ≤ 0.01) and a short duration of the diabetes was associated with reporting an improvement of body weight (OR 1.1 for each year less of diabetes, 95% CI 1.01-1.1, p ≤ 0.05).

At the time of follow-up, a reported improvement of the respective parameter at baseline was associated with reported improvements in the medical treatment (OR 3.0, 95% CI 1.5-6.0, p ≤ 0.01), improvement of GHb test results (OR 2.5, 95% CI 1.2-5.1, p ≤ 0.05), of body weight (OR 4.5, 95% CI 2.2-9.0, p ≤ 0.001), and of blood pressure (OR 2.9, 95% CI 1.2-6.8, p ≤ 0.05). Reporting an improvement of body weight at follow-up also was associated with a short duration of the diabetes (OR 1.1 for each year less of diabetes, 95% CI 1.01-1.135, p ≤ 0.05).

In sum, female patients, patients with a shorter duration of their diabetes (and presumably a lower severity of their type 2 diabetes), and patients with lower levels of depression more often regarded the DMP as beneficial at baseline. At follow-up, patients who had reported benefits at baseline still saw improvements.

### Attitude towards enrolment in a DMP

At baseline, 29% of the not enrolled patients said that they would like to participate in the DMP, whereas 59% said they would not like to do so. 12% of the not enrolled stated that they had never heard of the DMP (n = 323). The results from the follow-up interviews are similar: At follow-up 26% of the patients who were not enrolled at baseline and follow-up said that they would like to participate in the DMP, whereas 58% not. 16% of the not enrolled stated that they had never heard of the DMP (n = 141). A positive attitude towards enrolment was associated with higher education at baseline (OR 3.6, 95% CI 1.4-9.2, p ≤ 0.01). At follow-up those who had articulated a wish for enrolment at baseline more often reported this wish - again (OR 5.0, 95% CI 1.7-14.4, p ≤ 0.01).

## Discussion

In the view of patients with type 2 diabetes the German DMP improves parameters of process quality of care. Enrolled patients show a more frequent utilization of the services defined in the DMP: patient education courses for diabetes, regular encounters with the physician, referrals to the ophthalmologist and annual foot examinations. Also they more frequently possess a diabetes passport. The finding that more enrolled patients are mainly attended by a diabetologist can be interpreted as an indicator of process quality but also as an indicator of overusage of specialists. The German DMP only defines criteria for referral but does not intend to shift the main treatment responsibility to specialists. Except for referrals to the ophthalmologist and specialty of the attending physician the parameters statistically significant at baseline were also statistically significant one year later. These findings are in line with those of Szecsenyi et al. [15]. In their postal survey of 1.399 patients those enrolled in the DMP reported of a more structured care that reflected the core elements of the chronic care model than those not enrolled. They are also in line with an analysis of out-patient claims data of the Barmer Ersatzkasse, another German statutory health insurance, by Graf et al. [16]. They compared claims data of around 80.000 insurants who never participated in a DMP with around 81.000 insurants who were continuously enrolled since 2005. Among others the data revealed that enrolled patients had visited a patient training program for type 2 diabetes, had seen an ophthalmologist and a diabetologist more frequently than not enrolled patients.

With the higher frequency of these services one could expect an improvement in outcome variables. Research has shown varying evidence for the effectiveness of DMPs on outcomes depending on the outcome parameter in question. Patient education can improve therapy adherence and the ability to deal with the disease in every day life [17] as well as foster short-term improvements in GHb [18] and blood pressure levels [19]. Norris et al. forwarded strong evidence that disease management interventions are effective in improving glycaemic control [20]. Other studies with single-group, pre-post designs with 2-year follow-up also showed significant improvements in glycaemic control [21] and in the vascular risk profile (GHb, blood pressure and blood lipids) [22]. In contrast, diabetes passports do not seem to have much impact on outcome measures [23,24] and a Cochrane Review on adherence to treatment in patients with type 2 diabetes did not find significant effects of current inter-ventions [25]. The effect of disease management on quality of life has not been studied thoroughly yet [20,26].

In our study, however, no outcome differences between enrolled and not enrolled patients were found in the multivariate analyses, except for the risk of not knowing GHb test results at baseline being higher in the not enrolled group. These results must be interpreted with caution because we do not know whether there were differences between enrolled and not enrolled patients at the time of enrolment. Interestingly most of the outcome measures at follow-up were strongly associated with the respective outcome measures at baseline, which indicates a low impact of the DMP on changing outcomes within one year of follow-up. It is also possible, that the program duration of at maximum 3.5 years at the time of the interview was insufficient to demonstrate outcome quality differences. Another reason for the lack of outcome differences might be the complex nature of outcome indicators that are dependent on a great number of factors beyond the process quality of medical care, e.g. the sociodemographic situation. To sum up, indicators of process quality seem to be more easily changed by the DMP than indicators of outcome quality.

Despite the lack of outcome quality differences, 16 to 36% of the DMP participants reported that the medical treatment of their diabetes, GHb, blood pressure values and/or their body weight had improved due to enrolment. Especially female patients, patients with a shorter duration of their diabetes (and presumably a lower severity of their type 2 diabetes), and patients with lower levels of depression more often regarded the DMP as beneficial at baseline. At follow-up, patients who had reported benefits at baseline still saw improvements.

Negative effects of the DMP were reported rarely and were most frequent for an increase of body weight (in 10% of DMP participants at baseline and 12% at follow-up). Moreover, in the descriptive analyses, enrolled patients more often knew their GHb and blood pressure values. In sum, substantial outcome differences between enrolled and non-enrolled patients were not found in the multivariate analyses but around one third of the patients perceives a subjective outcome benefit of the DMP - unchanged within one year of follow-up.

Concerning the attitude towards enrolment around 60% of not enrolled patients at both interviews stated that they would not like to be enrolled. Hypotheses why patients do not wish to be enrolled mostly refer to the idea of "problem patients", i.e. not active and not motivated patients with a low therapy adherence. However, reasons for not-enrolment of patients have not yet been investigated in a methodologically sound manner.

The multivariate analyses revealed important confounders of enrolment in the DMP, which should always be controlled for in studies that analyse the effectiveness of the program. For example, patients with a longer duration of their diabetes, which might be an indicator for higher disease severity, show a more frequent utilization of the services defined in the DMP - independently of their enrolment status. Patients screened positive for depression show worse outcomes in subjective outcome indicators like self-rated health than patients without depression. Younger age is associated e.g. with a higher body-mass index and smoking, older age with better self-reported adherence to therapy, but lack of knowledge of GHb test results, when controlled for all other factors.

Strengths of our study are the multivariate analyses that controlled for important confounders of enrolment in the DMP, the interpretation of differences as statistically significant only if p ≤ 0.001 and the intention-to-treat analysis at follow-up. This leads to a conservative estimation of differences between enrolled and not enrolled patients and thus of the effects of the DMP. Therefore we regard the differences found in process measures as robust. In contrast to the analyses of Graf et al. [16] and Elkeles et al. [10,11], we also included type 2 diabetes patients who did not (yet) use any diabetes specific medication (15.8% of the enrolled and 19.4% of the not enrolled patients at baseline). Therefore, the results can be considered as valid for all patients with type 2 diabetes.

A limitation of our study is that considerably more not enrolled (72.7%) than enrolled patients (46.2%) could not be interviewed at baseline. Twice more not enrolled than enrolled patients had wrong contact data (N = 289 vs. 145) and three times more not enrolled patients refused to participate in the study (N = 325 vs. 110). Also 307 of the not enrolled patients in the baseline sample drawn by the insurance company denied to have diabetes although they had been identified by an ICD-10 code of E11, E12 or E14 in the insurance claims data. In enrolled patients these were only 14. The non-responder analysis revealed that the non-responders did not have a greater morbidity. In sum, for unknown reasons not enrolled patients were more difficult to recruit for this study. This might have biased the results. At follow-up, the same problem occurred to a lower degree: 30.2% of not enrolled patients could not be re-interviewed vs. 20.9% of the enrolled patients. Another weakness of our study is that the total sample included more men than women (61.5% men, p < 0.001). The low percentage of women in the whole sample is due to the historical structure of the GEK, which till the nineties was a special insurance company for craftsman (and their families). This specialty in the member structure of the GEK might limit the generalization of the results for all patients insured in the statutory health insurance in Germany. Additionally most enrolled and not enrolled patients we recruited had a high education level which indicates a selection bias in the direction of higher educated patient groups in our study. Against the background of evidence for a higher degree of education in enrolled patients [27] this bias might especially affect not enrolled patients. If this was true, the differences in process quality found are a conservative estimation as process quality is better for patients with higher education. Finally, another weakness of our study is that patient-reported data especially of clinical outcomes might not be valid. A collection of clinical data based on chart review would have been better, but was not realizable within this project. Therefore the lacking outcome differences between enrolled and not enrolled patients should not be interpreted in the sense of a non-effectiveness of the German DMP.

Some results of the descriptive analyses also deserve consideration. Mean GHb values in our study of 6.9% and blood pressure values of around 134/80 (both for the enrolled and the not enrolled) appear to be good. Concerning GHb they are comparable with the results of the DETECT study (6.9 ± 1.2%), a huge epidemiological german study. Regarding blood pressure the values in our study are even lower than in the DETECT study: 140.6 ± 18.3 mmHg [28]. However, the percentage of patients with an increased cardiovascular risk profile - defined as GHb ≥ 7.5% and ≥ 8.5% for age 75+ and/or blood pressure ≥ 140/90 mm Hg - is around 60% in all patients. This indicates that there is still a potential for improvement. The same applies to a percentage of around 17% smokers and around 35% of patients who report to be highly thirsty and/or suffer from frequent urination and/or from fatigue.

## Conclusions

In January 2009, the funding of the DMPs was reduced in the course an overall financial reform of the health insurance system in Germany. Statutory health insurances now have to decide whether to continue the DMP or to find another way of structuring care according to the needs of chronically ill patients. Our data suggest that due to the experiences of the patients the DMP for type 2 diabetes enhances process quality of diabetes care. It should not be withdrawn unless an evidently more promising approach is found.

## Competing interests

The authors declare that they have no competing interests.

## Authors' contributions

CK, HK and HvdB conceived and designed the study. IS, CK and HK coordinated it. HPB and BRT made important contributions to the acquisition of data. BG, IS and FH performed the statistical analyses. IS and HK drafted the manuscript, all other authors revised it critically. All authors read and approved the final manuscript.

## Pre-publication history

The pre-publication history for this paper can be accessed here:

http://www.biomedcentral.com/1472-6963/10/55/prepub
